# Rare Presentation of Partial Atrioventricular Septal Defect in the Elderly: A Case Report

**DOI:** 10.7759/cureus.89224

**Published:** 2025-08-01

**Authors:** Nana Osei, Abena K Agyekum, Christy Joseph, Samy I. McFarlane, Sabu John, Siddharth Dubey

**Affiliations:** 1 Internal Medicine, State University of New York Downstate Health Sciences University, Brooklyn, USA; 2 Cardiology, State University of New York Downstate Health Sciences University, Brooklyn, USA; 3 Internal Medicine/Endocrinology, Diabetes, and Metabolism, State University of New York Downstate Health Sciences University, Brooklyn, USA; 4 Cardiology, Kings County Hospital Center, Brooklyn, USA; 5 Pediatric Cardiology, Kings County Hospital Center, Brooklyn, USA

**Keywords:** atrial flutter, elderly, heart failure, partial atrioventricular septal defect, pulmonary edema

## Abstract

Partial atrioventricular septal defect (AVSD) is a congenital heart anomaly that typically manifests in childhood and rarely presents in elderly patients. However, this anomaly can lead to hemodynamically significant cardiopulmonary complications when left untreated. In this report, we present a case of a 68-year-old male patient who presented with symptoms of heart failure and atrial flutter and was found to have partial AVSD incidentally discovered by echocardiography. He was stabilized with diuretics and beta-blockers, and anticoagulation therapy was initiated, given his risk for thromboembolism as per his CHA2DS2VASc2 score. He was subsequently referred for surgical repair, which he declined, opting for medical therapy. The patient was regularly followed up as an outpatient with improvement of symptoms on medical therapy. This case highlights the severe complications of unrepaired partial AVSD in adulthood, highlighting the need for early diagnosis and intervention, since late identification in adulthood can result in significant morbidity. However, in cases where surgery is not possible or declined, patients can be adequately optimized medically to improve overall quality of life and decrease morbidity and mortality associated with this untreated anomaly.

## Introduction

Partial atrioventricular septal defect (AVSD) is a rare congenital heart anomaly, typically diagnosed and corrected in early childhood. It accounts for approximately 0.24 to 0.31 per 1000 live births, with partial forms being more common than complete defects [1, 2]. Partial AVSD is characterized by an ostium primum atrial septal defect (ASD) and a cleft in the anterior leaflet of the mitral valve, resulting in left-to-right shunting and progressive volume overload of the right heart chambers [1, 3]. Without early repair, these structural abnormalities may lead to complications such as arrhythmia, pulmonary hypertension, and congestive heart failure [4]. 

While pediatric detection and intervention have significantly improved outcomes, undiagnosed cases that persist into adulthood are uncommon, and presentation in late adulthood is exceedingly rare [5]. In such patients, lack of routine pediatric care or lack of symptoms early in life results in delayed diagnosis, which is often uncovered during evaluation for cardiac symptoms. Presentation beyond the sixth decade is exceedingly rare and poses unique diagnostic and management challenges [5, 6]. While early repair, ideally before the age of two, is associated with excellent outcomes, delayed surgical intervention in adults has shown favorable results, with significant symptomatic improvement and acceptable long-term survival [6-8].

We present the case of a 68-year-old man with previously undiagnosed partial AVSD who presented with acute decompensated heart failure. This case underscores the importance of considering congenital heart disease in the differential diagnosis of elderly patients with unexplained murmurs and heart failure, and it highlights the value of timely diagnosis and individualized management, even in late adulthood. 

## Case presentation

**Figure 1:**
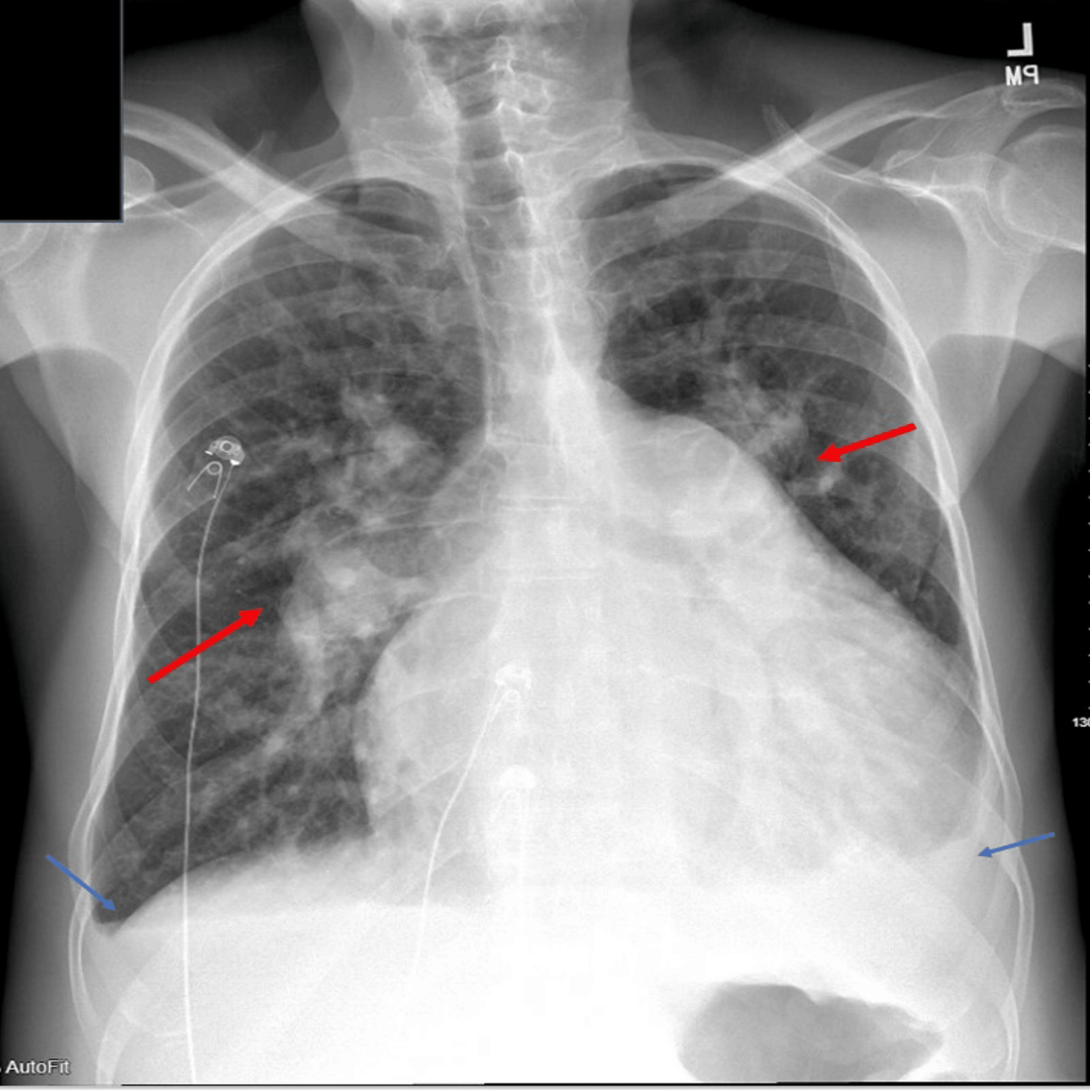
Chest X-ray showing pulmonary vascular congestion and cardiomegaly The red arrow points to areas of increased pulmonary vascular markings significant for pulmonary edema. Blue arrows show blunting of the costophrenic angle. This is significant for small bilateral pleural effusions.

**Figure 2:**
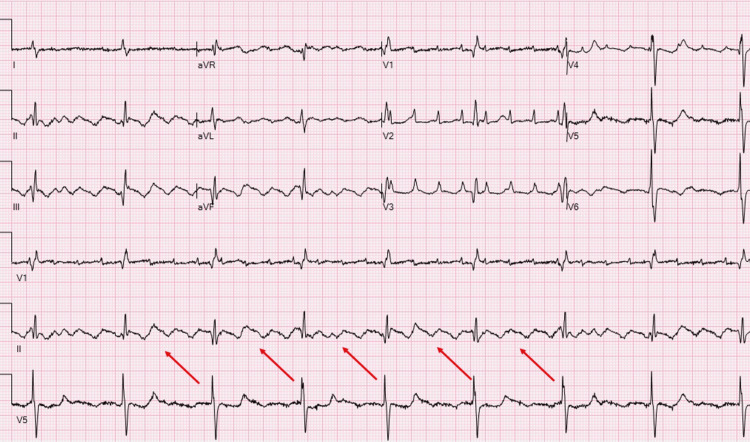
Electrocardiogram (ECG) showing atrial flutter The red arrow points to flutter waves that are evident in all the leads.

**Figure 3:**
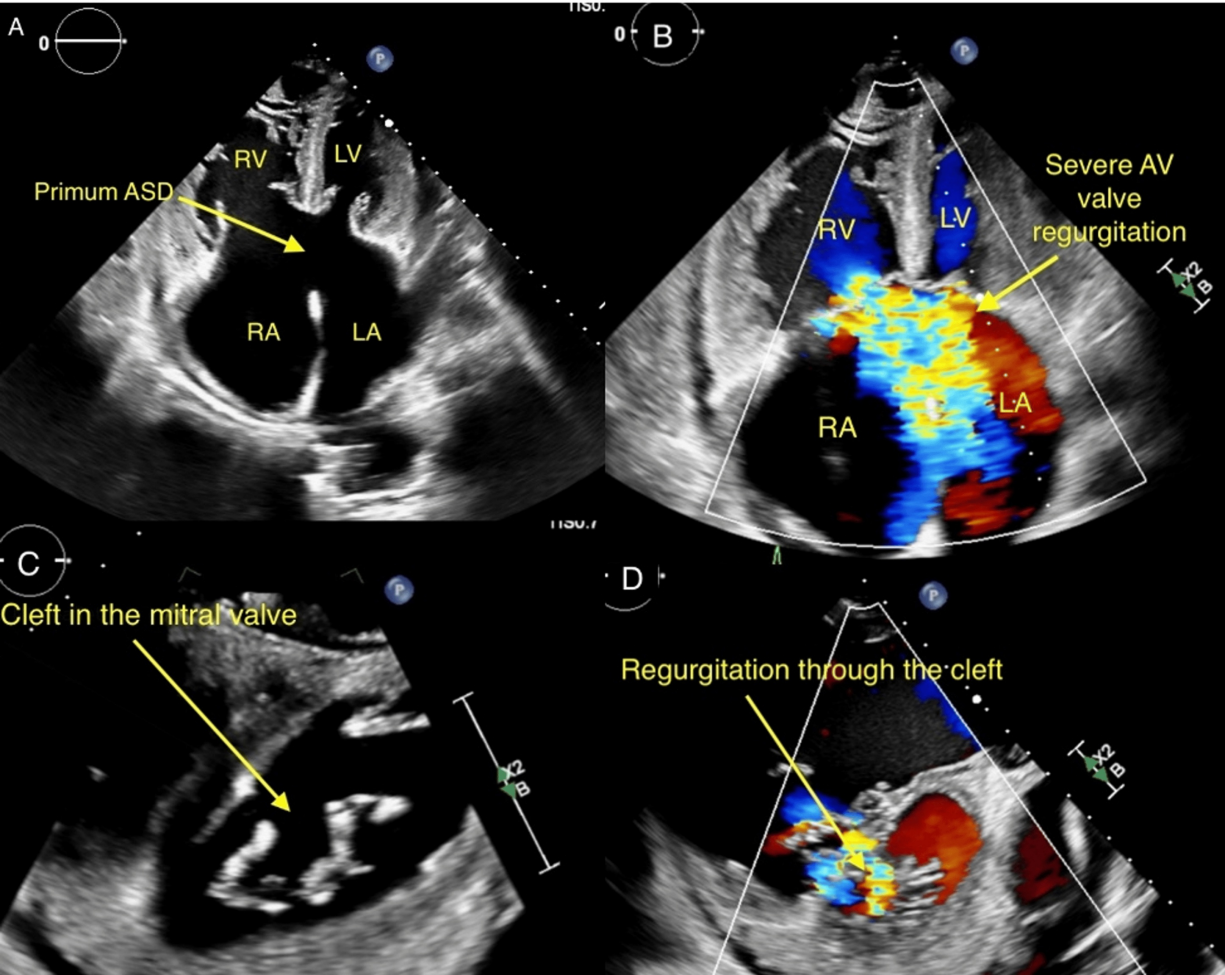
Transthoracic echocardiogram demonstrates (A) a partial AVSD with a large primum ASD; (B) Doppler shows severe left and right AV valve regurgitation; (C) There is a cleft in the anterior mitral leaflet; (D) Doppler shows severe regurgitation through the cleft. RV: right ventricle; LV: left ventricle; RA: right atrium; LA: left atrium; ASD: atrial septal defect; AV: atrioventricular; AVSD: atrioventricular septal defect

A 68-year-old man with previously undiagnosed partial AVSD presented with symptoms of shortness of breath, fatigue, and exercise intolerance. On physical examination, vitals were pertinent for heart rates in the 140s bpm, blood pressure of 138/93 mmHg, and oxygen saturation (SpO_2_) of 93% on room air, which improved to 100% on a 2-liter nasal cannula. The patient had jugular venous distension, a holosystolic murmur loudest at the left lower border with fixed split of S2, bibasilar crackles, and bipedal edema. Laboratory findings revealed an elevated pro-BNP of 10,996 pg/ml (Table 1), and a chest X-ray demonstrated pulmonary vascular congestion (Figure 1). Clinical presentation was consistent with acute heart failure. His electrocardiogram (ECG), depicted in Figure 2, showed atrial flutter with no ST-T wave changes. The echocardiogram demonstrated a partial AVSD with a large primum ASD and left-to-right shunt, shown in Figure 3. There was severe right atrioventricular valve regurgitation with severe dilation of the right-sided heart chambers. There was severe left-sided atrioventricular valve regurgitation, likely from the cleft in the anterior leaflet, with severe left atrial enlargement. There was moderate pulmonary hypertension and preserved left ventricular systolic function (LVEF) of 55%. He was admitted for further management and started on diuretics, beta-blockers, and anticoagulation as per his CHA2DS2VASc2 score, which led to clinical improvement. The patient was subsequently transferred for surgical repair. However, after multidisciplinary discussion, the patient declined surgery and opted for medical therapy. He was discharged in stable condition with guideline-directed medical therapy and has been maintained on regular outpatient follow-up. At one-year follow-up, his symptoms have remained well-controlled, and he continues to be clinically stable. 

**Table 1:** Laboratory values

Test	Result	Units	Reference range
pro-BNP	10,996	pg/mL	<125
Sodium	146	mmol/L	135-145
Potassium	3.6	mmol/L	3.5-5.1
Chloride	107	mmol/L	98-107
Carbon dioxide (CO_2_)	27	mmol/L	22-30
Blood urea nitrogen (BUN)	14.0	mg/dL	7-20
Creatinine	1.11	mg/dL	0.6-1.3
Glucose	88	mg/dL	70-100
Calcium	9.1	mg/dL	8.5-10.5
Albumin	3.9	g/dL	3.5-5.0
Total protein	6.6	g/dL	6.0-8.3
Total bilirubin	1.2	mg/dL	0.1-1.2
Alkaline phosphatase (ALP)	110	U/L	44-147
Alanine aminotransferase (ALT) (serum glutamic pyruvic transaminase (SGPT))	15	U/L	7-56
Aspartate aminotransferase (AST) (serum glutamic-oxaloacetic transaminase (SGOT))	20	U/L	10-40
Anion gap	12	mmol/L	8-16
Estimated glomerular filtration rate (eGFR)	>60	mL/min/1.73m²	>60
White blood cell (WBC)	3.90	10^3/uL	4.0-10.5
Red blood cell (RBC)	4.40	10^6/uL	4.5-5.9
Hemoglobin	13.4	g/dL	13.5-17.5
Hematocrit	41.3	%	40-52
Mean corpuscular volume (MCV)	93.9	fL	80-100
Mean corpuscular hemoglobin (MCH)	30.5	pg	27-33
Mean corpuscular hemoglobin concentration (MCHC)	32.4	g/dL	32-36
Mean platelet volume (MPV)	9.4	fL	7.5-11.5
Red cell distribution width (RDW)	15.6	%	11.5-14.5
Platelets	208	10^3/uL	150-450
Neutrophils %	55.4	%	40-60
Lymphocytes %	33.3	%	20-40
Monocytes %	7.9	%	2-8
Eosinophils %	2.3	%	1-4
Basophils %	0.8	%	0-1
Immature granulocytes %	0.3	%	0-0.4
Neutrophils abs	2.16	10^3/uL	1.5-8.0
Lymphocytes abs	1.30	10^3/uL	1.0-4.8
Monocytes abs	0.31	10^3/uL	0.2-0.8
Eosinophils abs	0.09	10^3/uL	0.0-0.5
Basophils abs	0.03	10^3/uL	0.0-0.2
Immature granulocyte abs	0.01	10^3/uL	0.0-0.2

## Discussion

Partial AVSD is typically diagnosed and repaired in childhood. However, as illustrated in this case, some patients may remain asymptomatic for decades and only present in late adulthood with signs of heart failure. In this patient, the previously unrecognized partial AVSD led to significant hemodynamic compromise, including chronic left-to-right shunting through the primum ASD and mitral regurgitation due to a cleft anterior mitral leaflet. This resulted in right heart volume overload, elevated pulmonary artery pressures, atrial enlargement, and atrial flutter, manifesting clinically as acute decompensated heart failure. A Qp:Qs ratio >1.5, as seen in this case, typically warrants surgical intervention due to the risk of irreversible pulmonary vascular disease and progressive cardiac dysfunction [9,10].

Surgical repair of partial AVSD involves patch closure of the ASD and repair of the mitral valve cleft [11]. While early repair, ideally by 18 months of age, is associated with optimal outcomes, delayed surgical intervention in adults has shown excellent survival and symptomatic improvement [6,12,13]. Platolla et al. reported a 10-year survival rate of over 90% in adult patients undergoing repair, with relatively low rates of reoperation or valve-related complications [5].

This case also emphasizes the importance of maintaining a high index of suspicion for congenital heart disease in older adults presenting with unexplained right heart dilation, murmurs, or new-onset heart failure. Management should address cardiopulmonary symptoms with diuretics and vasodilators, which reduce the preload and afterload [1]. Other comorbid conditions, such as atrial flutter, which occurs due to atrial stretching, should be managed accordingly. Advanced imaging, including echocardiography and cardiac MRI, plays a key role in confirming the diagnosis, quantifying shunt severity, and assessing surgical candidacy [2, 14].

There are limited reports of patients surviving into their seventh or eighth decades with unrepaired partial AVSD. One case report described an 80-year-old woman diagnosed later in life with a similar clinical presentation; however, she eventually experienced worsening symptoms on medical therapy alone and was deemed high-risk for surgical intervention [4]. The case highlights that while medical therapy may provide temporary stabilization, surgical correction is superior in terms of long-term survival and quality of life [6, 15].

Our case reinforces that even in the absence of a childhood diagnosis, timely identification of partial AVSD in adults and referral for surgical evaluation can lead to meaningful clinical improvement. In patients who decline surgery or are deemed inoperable, ongoing surveillance and symptom-guided medical management remain essential.

## Conclusions

Partial AVSD is a rare congenital heart condition that may remain undiagnosed into adulthood. While early surgical repair remains the standard of care and generally yields excellent long-term outcomes, diagnosis in elderly patients warrants individualized assessment of surgical candidacy and expected benefit, particularly in the context of age-related comorbidities and anatomical factors. In patients who decline or are deemed unfit for surgery, medical therapy may offer symptomatic relief but does not prevent long-term complications. Multidisciplinary team evaluation, including adult congenital cardiologists, imaging specialists, and cardiac surgeons, is essential to guide management. Longitudinal imaging plays a central role not only in diagnosis but also in monitoring for progression and valvular dysfunction in patients managed conservatively.
